# Electrophysiological evidence for increased auditory crossmodal activity in adult ADHD

**DOI:** 10.3389/fnins.2023.1227767

**Published:** 2023-08-29

**Authors:** Mia Schramm, Tatiana Goregliad Fjaellingsdal, Behrem Aslan, Paul Jung, Silke Lux, Marcel Schulze, Alexandra Philipsen

**Affiliations:** ^1^Department of Psychiatry and Psychotherapy, University of Bonn, Bonn, Germany; ^2^Department of Neurology, University of Lübeck, Lübeck, Germany; ^3^Department of Psychology, University of Lübeck, Lübeck, Germany; ^4^Center of Brain, Behavior and Metabolism (CBBM), University of Lübeck, Lübeck, Germany

**Keywords:** attention deficit and hyperactivity disorder (ADHD), sensory processing, EEG, crossmodal activity, auditory, visual

## Abstract

**Background:**

Attention deficit and hyperactivity disorder (ADHD) is a neurodevelopmental disorder characterized by core symptoms of inattention, and/or impulsivity and hyperactivity. In order to understand the basis for this multifaceted disorder, the investigation of sensory processing aberrancies recently reaches more interest. For example, during the processing of auditory stimuli comparable low sensory thresholds account for symptoms like higher distractibility and auditory hypersensitivity in patients with ADHD. It has further been shown that deficiencies not only exist on an intramodal, but also on a multimodal level. There is evidence that the visual cortex shows more activation during a focused auditory task in adults with ADHD than in healthy controls. This crossmodal activation is interpreted as the reallocation of more attentional resources to the visual domain as well as deficient sensory inhibition. In this study, we used, for the first time, electroencephalography to identify a potential abnormal regulated crossmodal activation in adult ADHD.

**Methods:**

15 adult subjects with clinically diagnosed ADHD and 14 healthy controls comparable in age and gender were included. ERP components P50, P100, N100, P200 and N200 were measured during the performance of a unimodal auditory and visual discrimination task in a block design. Sensory profiles and ADHD symptoms were assessed with inattention as well as childhood ADHD scores. For evaluating intramodal and crossmodal activations, we chose four EEG channels for statistical analysis and group-wise comparison.

**Results:**

At the occipital channel O2 that reflects possible crossmodal activations, a significantly enhanced P200 amplitude was measured in the patient group. At the intramodal channels, a significantly enhanced N200 amplitude was observed in the control group. Statistical analysis of behavioral data showed poorer performance of subjects with ADHD as well as higher discrimination thresholds. Further, the correlation of the assessed sensory profiles with the EEG parameters revealed a negative correlation between the P200 component and sensation seeking behavior.

**Conclusion:**

Our findings show increased auditory crossmodal activity that might reflect an altered stimulus processing resource allocation in ADHD. This might induce consequences for later, higher order attentional deployment. Further, the enhanced P200 amplitude might reflect more sensory registration and therefore deficient inhibition mechanisms in adults with ADHD.

## 1. Introduction

Attention deficit and hyperactivity disorder (ADHD) is a neurodevelopmental disorder that is characterized by inappropriate levels of inattention, hyperactivity, and/or impulsivity (American Psychiatric Association, 2013). Although it was often seen as a disorder that only affects children, around 60% of cases report symptom persistence in adulthood (Sibley et al., 2017). Whereas hyperactivity and impulsivity are often the main symptoms in children and decrease with increasing age, adults are mostly affected by inattention (Uchida et al., 2015). While ADHD research mainly focuses on the core symptoms and their neuronal underpinnings, the basic and important area of reception and processing of stimuli is rarely examined (Schulze et al., 2020).

Sensory processing describes the nervous systems ability to receive, modulate, integrate, and organize external stimuli in order to generate an appropriate response to the environment (Hebert, 2015). There is evidence that sensory processing of several modalities in people with high scores in the adult ADHD self-report scale (ASRS) is deficient compared to those with low ASRS scores (Panagiotidi et al., 2018). Sensory processing of adults with ADHD is marked by higher scores in sensory sensitivity, low registration, and sensory avoiding behavior. At the same time, sensation seeking behavior scores are found to be either higher or lower (Bijlenga et al., 2017; Kamath et al., 2020). Modality specific impairment in sensory processing have mainly been reported for vision and audition (Schulze et al., 2020). On the behavioral level, patients report an auditory hypersensitivity which is reflected in higher distractibility from sounds, e.g., the inability to suppress background noises like a ticking clock and overinclusion of auditory stimuli (Faraone et al., 2000; Panagiotidi et al., 2017). Visual processing has shown to be deficient as well, indicated by worse perception of depth and higher distractibility (Panagiotidi et al., 2017), worse temporal allocation of visual attention (Hollingsworth et al., 2001) and slower response times in a compound search task (Cross-Villasana et al., 2015).The underlying principles of these perceptional sensitivities are far from understood, but evidence is pointing to inhibitory and modulatory deficits at early stimulus processing capacities (Micoulaud-Franchi et al., 2019).

Early stimulus processing can be measured electrophysiologically, for example, by means of event-related potentials (ERPs; Barry et al., 2009). For instance, the P200 component that is the peak positivity that occurs between 175 and 250 ms after a stimulus onset, is associated with early sensory processing steps including like stimulus registration, encoding, evaluation, and discrimination, early attentive mechanisms and selective attention, as well as the inhibition of further processing of competing information (Näätänen, 1992; Korostenskaja et al., 2008; Barry et al., 2009; Lazzaro et al., 2009; Sur and Sinha, 2009; Stefanou et al., 2020). This component has been reported to be deficient in patients with ADHD, showing an upregulation (Barry et al., 2009).

When a person, neurotypical as well as on the ADHD spectrum, is confronted with stimuli of a certain sensory modality (e.g., auditory information), the other senses are also co-activated (e.g., visual cortices; Molholm et al., 2002; McDonald et al., 2013; Hillyard et al., 2016). This co-activation, also called crossmodal activation has been reported in several modalities, especially between the auditory and visual domain (Hillyard et al., 2016). It is well documented that primary auditory and visual cortices are anatomically connected and influence each other already at early stages of sensory processing (Falchier et al., 2002; Molholm et al., 2002; Schroeder et al., 2003; Komura et al., 2005). For example, Hillyard et al. (2016) reported improved detection and discrimination of a visual stimulus resulting from a preceding auditory tone (Korostenskaja et al., 2008).

These audiovisual perceptual modulations further correlate with EEG phenomena, such as the auditory-evoked contralateral occipital positivity (ACOP). It describes a net positivity that occurs in the contralateral occipital electrodes 250–400 ms after the presentation of a sound. A recent study shows that the ACOP reflects facilitated visual processing and therefore might indicate an involuntary reallocation of attentional resources to the visual domain, as proposed by Hillyard et al. (2016) (Keefe et al., 2021).

Recent evidence points to the fact that crossmodal sensory processing might be deficient in people with ADHD (Salmi et al., 2018). Salmi et al. (2018) examined auditory and visual attentional mechanisms in adults with ADHD using fMRI and measured a higher crossmodal visual activation in a unimodal focused auditory attention task compared to controls. However, given the low temporal resolution of fMRI, it is difficult to disentangle whether those activations are the result of early sensory processing or attentional mechanisms. Therefore, in the current study, we used, for the first time, electroencephalography to identify a potential abnormal regulated crossmodal activation in adult ADHD. To provoke a potential crossmodal response for unisensory stimuli, we performed an auditory and visual discrimination task separately. Since prior research shows deficient inhibitory activity at early sensory EEG components like P50, P100, and P200, we assume crossmodal spreading of sensory information. We hypothesize that adults with ADHD show higher auditory to visual crossmodal activations.

## 2. Methods

### 2.1. Participants

15 patients [five female; age (M ± SD) 34 ± 7.1 years] with clinically diagnosed ADHD, based on the Diagnostic and Statistical Manual of Mental Disorders (Heinzl, 2018), and 14 healthy controls with comparable age and gender distribution [five female; age (SD) 32.14 ± 9.93 years] were recruited through the university hospital’s psychiatric outpatient department or by using bulletin boards. The diagnosis of ADHD was given by a specialized psychiatric consultant after a detailed diagnostic interview with consideration of the patients’ psychiatric and developmental history, observer reports as well as somatic and psychiatric differential diagnosis. Patients with psychiatric disorders such as bipolar disorder, obsessive compulsive disorder, panic disorder, or neurological disorders were excluded from this study. Patients who were taking stimulant medication (e.g., Methylphenidate) were asked to discontinue those at least 24 h before the experiments. All participants gave full written consent to participate in this study. The study was approved by the medical faculty’s ethics committee of the University of Bonn.

Attention deficit and hyperactivity disorder symptoms were additionally investigated by the following questionnaires: The Conners Adult ADHD Rating Scales (CAARS, long version, self-rated; Conners et al., 1999) to assess current ADHD symptoms, the Wender Utah Rating Scale (WURS-k; Ward et al., 1993) to rate retrospectively ADHD symptoms in childhood. Additionally, the Beck Depression inventory (Beck et al., 1961) and the Adult Sensory Profile (ASP; Brown et al., 2001) were administered to investigate current depressive symptoms and sensory behavior.

### 2.2. Stimuli and task

The experiment was programmed and presented using Presentation® software (Version 18.0, Neurobehavioral Systems, Inc., Berkeley, CA, United States, www.neurobs.com) and digitized using Labstreaminglayer (LSL; https://github.com/sccn/labstreaminglayer).

#### 2.2.1. Auditory discrimination task

The paradigm was adapted from Haigh et al. (2016). In the auditory task, we used sine wave tones with a duration of 20 ms and varying frequencies, presented via over ear headphones with individual adjusted sound pressure levels. The first presented tone of each trial had a fixed frequency at 1,100 Hz, whereas the second tone’s frequency varied between 2,100 and 10 Hz, depending on the participants’ responses.

**Figure 1:**
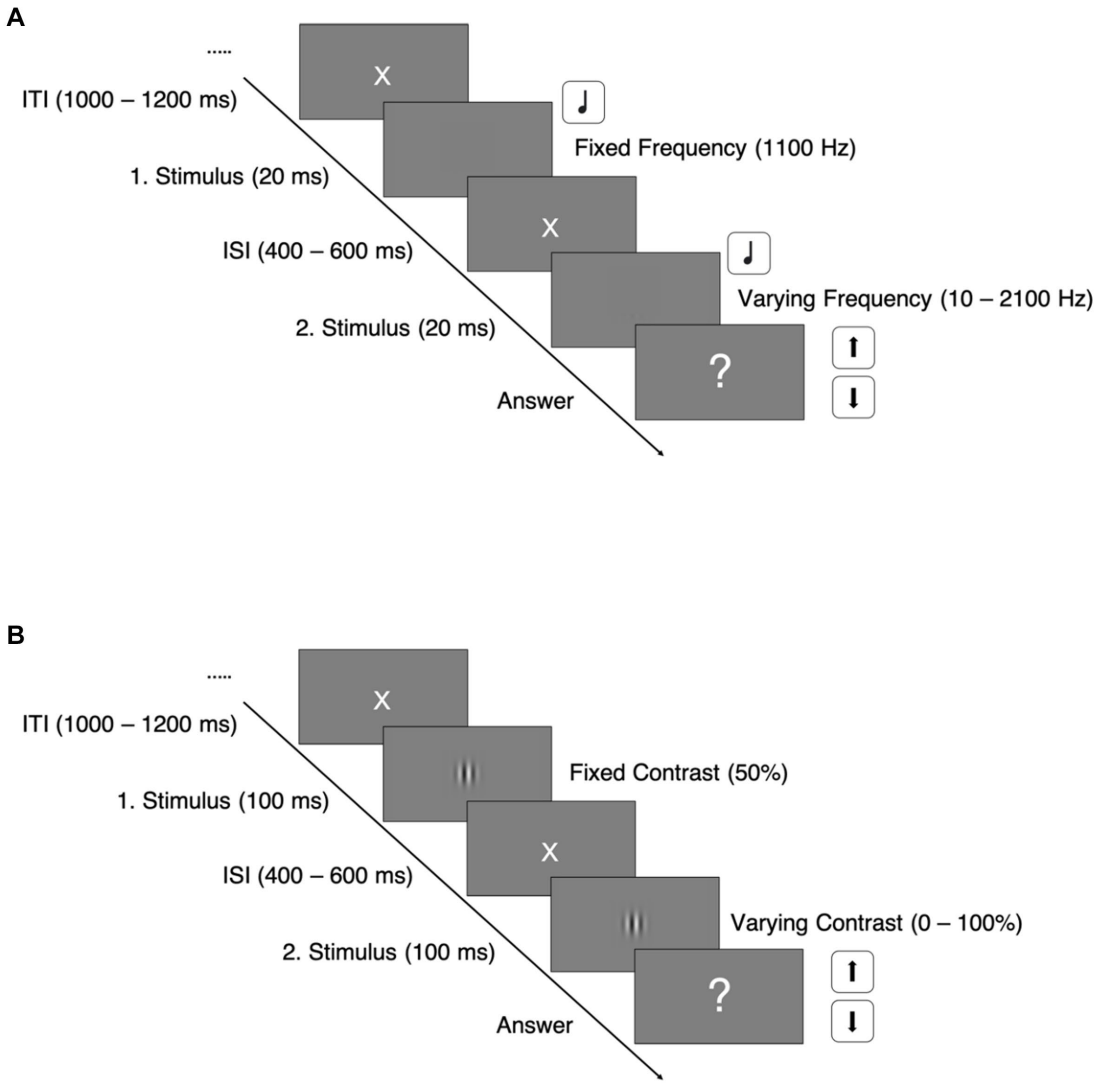
Paradigm trial sequences. ITI, intertrial interval; ISI, interstimulus interval; **(A)** Auditory discrimination task; **(B)** Visual discrimination task.

Every trial sequence started off with a white central fixation cross on a gray background for 1,000–1,200 ms. It was followed by the presentation of the two auditory stimuli, which were separated by an interstimulus interval for 400–600 ms (See Figure 1A).

Immediately after the second stimulus’ offset, the participant was asked to decide as fast as possible whether the *second* stimulus’ frequency was higher or lower than the first one by pressing the arrow up or down button on a keyboard.

Two interleaved three-down one-up staircases with fixed steps and opposite extremes as starting points were used to derive each individual’s discrimination threshold as well as to keep their attention.

One staircase started with the highest possible frequency interval between the two stimuli whereas the other staircase started with the minimal frequency interval. After being correct for three consecutive trials at a particular contrast, the interval decreased and increased after one false answer in fixed 10 Hz steps. Total discrimination thresholds were calculated by averaging the separately extracted minimal thresholds for each stair case.

#### 2.2.2. Visual discrimination task

Trial sequences of the visual task were structured equally to the auditory task (see Figure 1B). In the visual discrimination task, we used vertically oriented Gabor patches with varying contrasts, shown for 100 ms. The first Gabor patch of every trial had a fixed contrast at 50%, whereas the contrast of the second patches varied between 0 and 100%, also depending on the participants’ response and adjusted by the same staircase mechanism: The staircases started at either 0.5 or 50% contrast difference and adjusted the difference in fixed steps of 0.5% contrast difference.

#### 2.2.3. Procedure

Each session lasted 2 h and took place in a dimmed room, where participants were seated in front of a computer monitor at 60 cm distance and equipped with headphones (Sennheiser HD300 Pro) for the auditory task.

Participants performed the auditory and visual discrimination task in a block design with one block per task in a pseudorandomized order. Each task consists of 350 trials and started with a training of 10 trials.

### 2.3. EEG recording environment

We recorded EEG with a mobile system (Smarting mBrainTrain, Belgrade, Serbia) with 22 pre-mounted Ag/AgCl electrodes (Easycap GmbH, Herrsching, Germany) that were placed according to the international 10/20 System (Fp1, Fp2, F3, F4, F7, F8, Fz, C3, Cz, C4, T7, T8, CPz, P3, Pz, P4, P7, P8, O1, and O2). The ground (AFz) and reference electrode (FCz) were embedded in the fronto-polar region and in the frontocentral region, respectively.

EEG data were digitized with a wireless EEG amplifier at a sampling rate of 500 Hz. Impedances were kept below 10 kΩ.

### 2.4. EEG analysis

Data were analyzed with EEGLAB (Delorme et al., 2011) in Matlab (version 2021b; MATLAB—MathWorks, 2022). For preprocessing, we adapted the protocol of Fjaellingsdal et al. (2016).

For artifact attenuation, an Independent Component Analysis (ICA) was computed on the merged blocks of visual and auditory presentation. Prior to ICA, the data were high-pass filtered at 1 Hz and low-pass filtered at 60 Hz (FIR filter, window type “Hann,” cutoff frequency − 6 dB). Dummy epochs of 1 s that were unrelated to the task structure were generated and an automatic epoch rejection was applied whenever two standard deviations of the mean signal were exceeded. ICA weights were saved on the raw data.

For ERP analysis, the raw data sets were high-pass filtered at 0.1 Hz and low-pass filtered at 30 Hz (FIR filter, window type “Hann,” cutoff frequency − 6 dB). Artifactual ICA components were identified by visual inspection and confirmed by the component classification plugin ICLabel (Pion-Tonachini et al., 2019). After component rejection, data were re-referenced to the arithmetic mean of the left and right mastoid (T7, T8). Epochs were generated relative to the onset of the visual stimulus/auditory stimulus from −100 to 300 ms and baseline corrected from −100 to 0 ms.

### 2.5. Statistical analysis

Demographical data of the two groups were compared by using independent samples *t*-tests (Table 1). For statistical analysis of the auditory intramodal activation, channels Fz and Cz (Näätänen and Picton, 1987; Plourde, 2006; Snyder et al., 2006) were selected. Expecting a posterior distribution of auditory to visual crossmodal activations, channels O1 and O2 (Odom et al., 2004) were selected for statistical analysis. ERP responses to the first stimulus only were selected for analysis.

**Table 1:** Demographical data row values of different self-rating questionnaires are given in means (M) and standard deviations (SD).

	Patients (*n* = 15; 5 f)	Controls (*n* = 14; 5 f)
Age (years), M (SD)	34 (10.72)	32.14 (9.94)
WURS-k (ADHD symptomatology in childhood)^**^	36.45 (13.58)	13.50 (12.51)
BDI-II (Depression scores)	9.14 (11.29)	3.93 (3.34)
CAARS (ADHD symptomatology)		
Inattention scores^**^	21.47 (5.53)	7.29 (3.65)
Hyp./Imp. scores^**^	21.93 (5.18)	7 (4.32)
Medication		
Methylphenidate (*n*)	9	-
Bupropion (*n*)	1	-
Adult Sensory Profile		
Low registration^**^	38.20 (6.20)	27.14 (6.24)
Sensory avoidance^*^	42.27 (9.87)	35.07 (7.03)
Sensation seeking	47.00 (7.33)	49.36 (7.58)
Sensory sensitivity^*^	42.60 (9.22)	35.93 (6.91)

*n*, number; *f*, female; *CAARS*, Conners adult ADHD rating scales, *Hyp./Imp.*, hyperactivity/impulsivity; *WURS-k*, Wender-Utah rating scale; *BDI*, beck depression inventory. Group differences (^*^p < 0.05; ^**^p < 0.01) were calculated with independent sample *t-*test.

**Figure 2:**
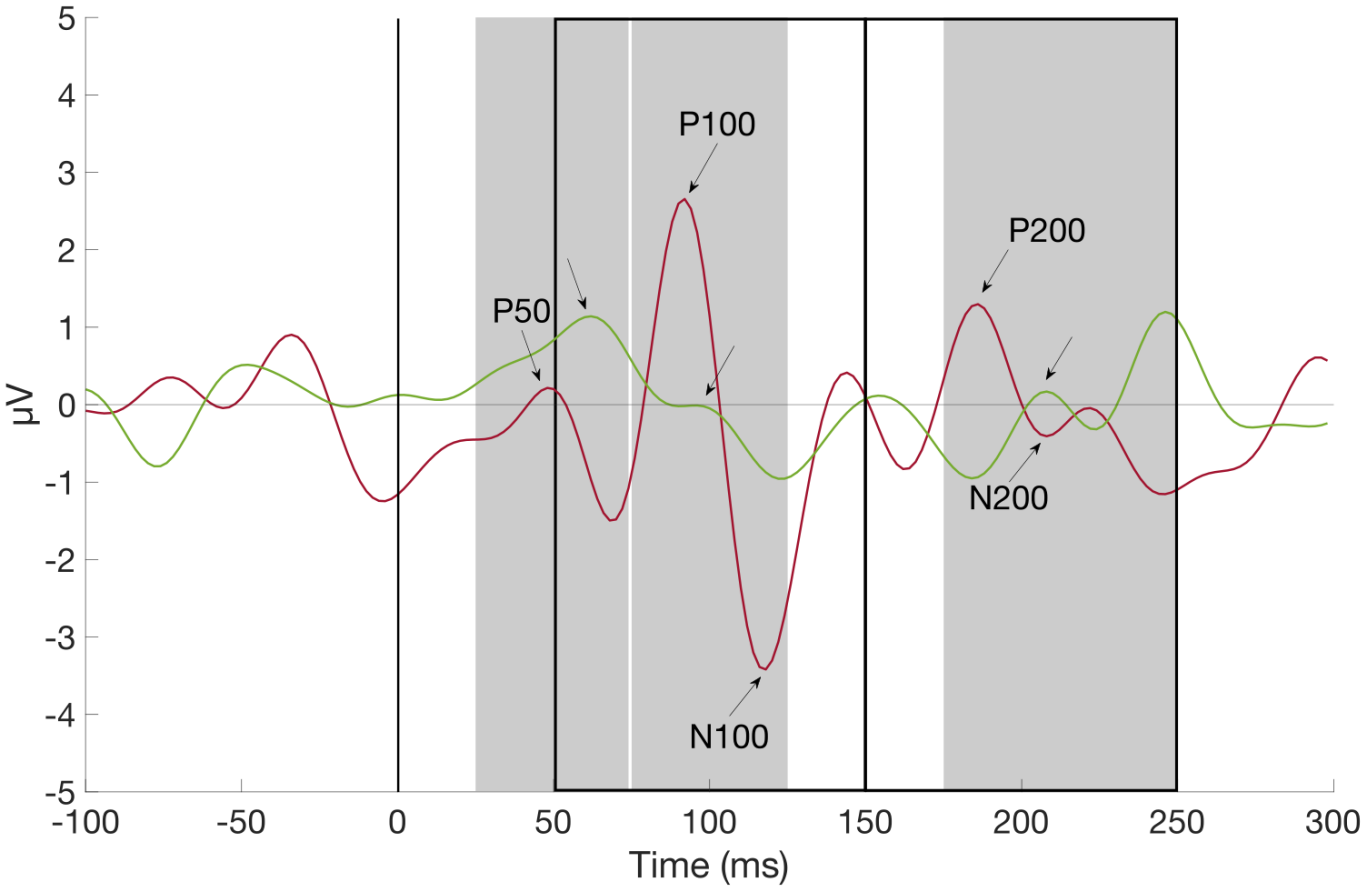
Grand average waveform of P50, P100, N100, P200, and N200 at channel O2, ADHD (red) vs. Control Subject (green). First auditory stimulus was presented at time zero. Component amplitudes of P50, N100, P100, N200, and P200 were identified by detecting peaks in pre-defined time windows (gray rectangles for positive, black rectangles for negative components): Between 25 and 74 ms (P50), 75 and 125 ms (P100), 50 and 150 ms (N100), 175 and 250 ms (P200), and 150 and 250 ms (N200) after stimulus onset (black line), respectively.

Event-related potential components were stimulus locked to the first presented tone/gabor patch and identified as the most positive deflections 25–74 ms (P50), 75–125 ms (P100), and 175–250 ms (P200) as well as the most negative deflections 50–150 ms (N100) and 150–250 ms (N200) relative to stimulus onset by using the MATLAB function “findpeaks” (part of the Matlab Signal Processing Toolbox; see Figure 2). The selected component amplitudes were then group-wise compared with independent samples t-tests (see Tables 2, 3).

**Table 2:** Results auditory discrimination task—analysis and group-wise comparison of intra- and crossmodal EEG parameters.

Cz (Intramodal)				
	Patients	Controls		
	M (SD)	M (SD)	*t*	*p*
p50	1.12 (1.66)	0.90 (1.21)	0.41	0.684
p100	1.02 (0.92)	0.63 (1.22)	0.965	0.343
n100	−3.31 (2.61)	−3.58 (2.31)	0.279	0.782
p200	4.49 (2.84)	4.12 (2.97)	0.348	0.730
n200	−0.19 (1.53)	0.67 (1.67)	−1.405	0.171
Fz (Intramodal)				
	Patients	Controls		
	M (SD)	M (SD)	*t*	*p*
p50	1.12 (1.66)	1.18 (1.24)	−0.104	0.918
p100	1.27 (1.17)	0.98 (1.45)	0.587	0.562
n100	−2.29 (2.38)	−4.18 (2.44)	2.037	0.052
p200	4.69 (2.27)	4.97 (2.14)	−0.350	0.728
n200	−1.08 (1.81)	0.76 (2.61)	−**2.312**	**0.041**
O1 (Crossmodal)				
	Patients	Controls		
	M (SD)	M (SD)	*t*	*p*
p50	0.83 (1.37)	0.89 (0.88)	−0.140	0.890
p100	0.72 (0.86)	0.27 (0.85)	1.420	0.167
n100	−1.64 (1.93)	−1.29 (0.66)	−0.627	0.536
p200	1.00 (0.71)	0.61 (0.64)	1.568	0.128
n200	−0.72 (0.42)	−0.48 (0.65)	−1.133	0.267
O2 (Crossmodal)				
	Patients	Controls		
	M (SD)	M (SD)	*t*	*p*
p50	0.76 (1.25)	1.00 (0.90)	−0.595	0.557
p100	0.82 (0.82)	0.41 (0.62)	1.537	0.136
n100	−1.57 (1.73)	−1.40 (0.76)	−0.319	0.752
p200	1.22 (0.73)	0.68 (0.48)	**2.341**	**0.027**
n200	−0.47 (0.66)	−0.50 (0.53)	0.152	0.880

Mean amplitudes with standard deviations (in parenthesis) of the auditory discrimination task at channels *C*_z_, Fz, O1, and O2. Significant results are highlighted in bold.

**Table 3:** Results visual discrimination task—analysis and group-wise comparison of intra- and crossmodal EEG parameters.

Cz (Crossmodal)				
	Patients	Controls		
	M (SD)	M (SD)	*t*	*p*
p50	0.27 (0.61)	0.29 (0.62)	−0.099	0.922
p100	0.77 (1.78)	−0.37 (1.59)	1.818	0.080
n100	−1.48 (1.34)	−2.80 (2.19)	1.905	0.068
p200	3.47 (2.92)	3.13 (2.24)	0.348	0.730
n200	0.45 (2.18)	−0.23 (2.24)	0.799	0.431
Fz (Crossmodal)				
	Patients	Controls		
	M (SD)	M (SD)	*t*	*p*
p50	0.31 (0.59)	0.31 (0.59)	0.654	0.259
p100	0.00 (0.86)	−0.18 (0.64)	0.587	0.562
n100	−1.59 (1.31)	−3.19 (1.64)	**2.801**	**0.009**
p200	2.70 (2.70)	2.46 (2.10)	0.260	0.797
n200	−0.53 (0.84)	−0.89 (2.07)	0.595	0.557
O1 (Intramodal)				
	Patients	Controls		
	M (SD)	M (SD)	*t*	*p*
p50	0.56 (1.22)	2.30 (2.69)	−**2.247**	**0.033**
p100	2.34 (2.25)	2.54 (2.37)	−0.222	0.826
n100	−0.37 (1.30)	−0.46 (1.34)	0.177	0.861
p200	2.27 (2.56)	2.83 (1.92)	−0.660	0.515
n200	0.09 (1.64)	0.71 (1.72)	−0.973	0.339
O2 (Intramodal)				
	Patients	Controls		
	M (SD)	M (SD)	*t*	*p*
p50	0.40 (1.10)	1.92 (2.44)	**−2.200**	**0.037**
p100	2.33 (2.05)	2.18 (2.20)	0.947	0.855
n100	−0.95 (0.90)	−0.39 (1.81)	−1.031	0.312
p200	2.34 (1.76)	3.31 (1.80)	0.549	0.158
n200	0.28 (1.14)	0.78 (1.88)	−0.842	0.407

Mean amplitudes with standard deviations (in parenthesis) of the auditory discrimination task at channels *C*_z_, Fz, O1, and O2. Significant results are highlighted in bold.

Behavioral data including response times, response times only when a correct answer was given (Hit only) as well as hit/miss-rates were also group-wise compared with independent samples *t*-tests (see Tables 4, 5). In order to evaluate error rates throughout the experiment between the groups, miss rates between the first 170 trials and the second 170 trials were compared.

**Table 4:** Behavioral data of the auditory discrimination task.

	Patients	Controls		
	M (SD)	M (SD)	*t*	*p*
Response time (ms)	614.44 (134.90)	598.74 (108.63)	0.344	0.734
Response time hits (ms)	593.53 (119.95)	578.76 (99.79)	0.359	0.722
Thresholds audio (Hz)	38.67 (47.79)	13.57 (7.19)	1.942	0.063
Trials to threshold (*n*)	172.47 (65.40)	142.86 (43.43)	1.425	0.166
Trials to threshold (%)	49.28 (18.69)	40.82 (12.41)	1.425	0.166
Hits (%)	80.86 (7.70)	86.01 (6.52)	−1.961	0.060
Misses (%)	19.14 (7.68)	13.99 (6.44)	1.961	0.060
Misses 1st half (%)	16.68 (1.36)	11.99 (1.50)	**2.11**	**0.044**
Misses 2nd half (%)	29.06 (18.18)	10.83 (2.11)	1.80	0.083

*SD*, standard deviation; *t* and *p* values calculated with independent sample *t*-tests.The ADHD group made significantly more mistakes in the first half of the task compared to the healthy controls. Significant results are highlighted in bold.

**Table 5:** Behavioral data of the visual discrimination task.

	Patients	Controls		
	M (SD)	M (SD)	*t*	*p*
Response time (ms)	496.52 (130.73)	468.80 (149.99)	0.532	0.599
Response time hits (ms)	482.03 (108.71)	460.59 (138.10)	0.466	0.645
Thresholds visual (Hz)	13.70 (11.10)	14.00 (10.28)	−0.075	0.940
Trials to threshold (*n*)	153.93 (96.69)	148.71 (82.64)	0.156	0.877
Trials to threshold (%)	43.98 (27.63)	42.49 (23.61)	0.156	0.877
Hits (%)	72.84 (2.27)	73.13 (1.83)	−0.373	0.712
Misses (%)	27.16 (2.42)	26.87 (1.68)	0.466	0.645
Misses 1st half (%)	22.52 (0.73)	20.54 (0.55)	0.343	0.734
Misses 2nd half (%)	25.41 (0.71)	23.25 (0.47)	0.294	0.770

*SD*, standard deviation; *t* and *p* values calculated with independent sample *t*-tests.

Before statistical comparison, response times had been outlier corrected with the MATLAB function “rmoutliers” with an outlier being defined as value above 3 median absolute deviation (MAD). Additionally, we correlated EEG amplitudes of the patient group with their ASP scores using the Pearson’s Correlation Coefficient. All statistical analysis was performed using SPSS Statistics (Version 28.0.1.1).

## 3. Results

### 3.1. Demographical data and questionnaires

Demographical data of the 15 adult subjects with ADHD and 14 healthy controls are shown in Table 1. There was no significant group difference for age [M_ADHD_ *= 34 (SD = 10.72),* M_Controls_ = 32.14 (SD = 9.94), *t =* 0.483, *p* = 0.633] or gender [*X^2^* (1, *N* = 29) = 0.082, *p* = *0.797*].

Conners Adult ADHD Rating Scales inattention and hyperactivity/impulsivity scores (*t =* 8.1, *p* < 0.01) as well as WURS-k scores (*t =* 4.39, *p* < 0.01) are significantly higher in the patient group, which is in line with their clinically confirmed ADHD diagnosis. Further, nine patients took regular ADHD medication with the agent methylphenidate (MPH) that was paused at least 24 h before the experiment.

Although BDI scores reflected descriptively more depressive symptoms in the patient group, no significant difference between groups was found (*t =* 1.66, *p* = 0.109).

Results of the ASP questionnaire show that adults with ADHD scored significantly higher in the domains low registration [M_ADHD_ *= 38.2 (SD = 6.2),* M_Controls_ = 27.1 (SD = 6.2), *t =* 4.79, *p* < 0.01], sensory avoidance [M_ADHD_ *= 42.3 (SD = 9.9),* M_Controls_ = 35.1(SD = 7.0), *t* = 2.25, *p* = 0.03], and sensory sensitivity [M_ADHD_ *= 42.6 (SD = 9.2),* M_Controls_ = 35.9 (SD = 6.9), *t* = 2.19, *p* = 0.03], but not in the domain sensation seeking behavior [M_ADHD_ *= 47.0 (SD = 7.3),* M_Controls_ = 49.4 (SD = 7.6), *t* = − 0.85, *p* = 0.4].

### 3.2. Behavioral data

#### 3.2.1. Auditory discrimination task

**Figure 3:**
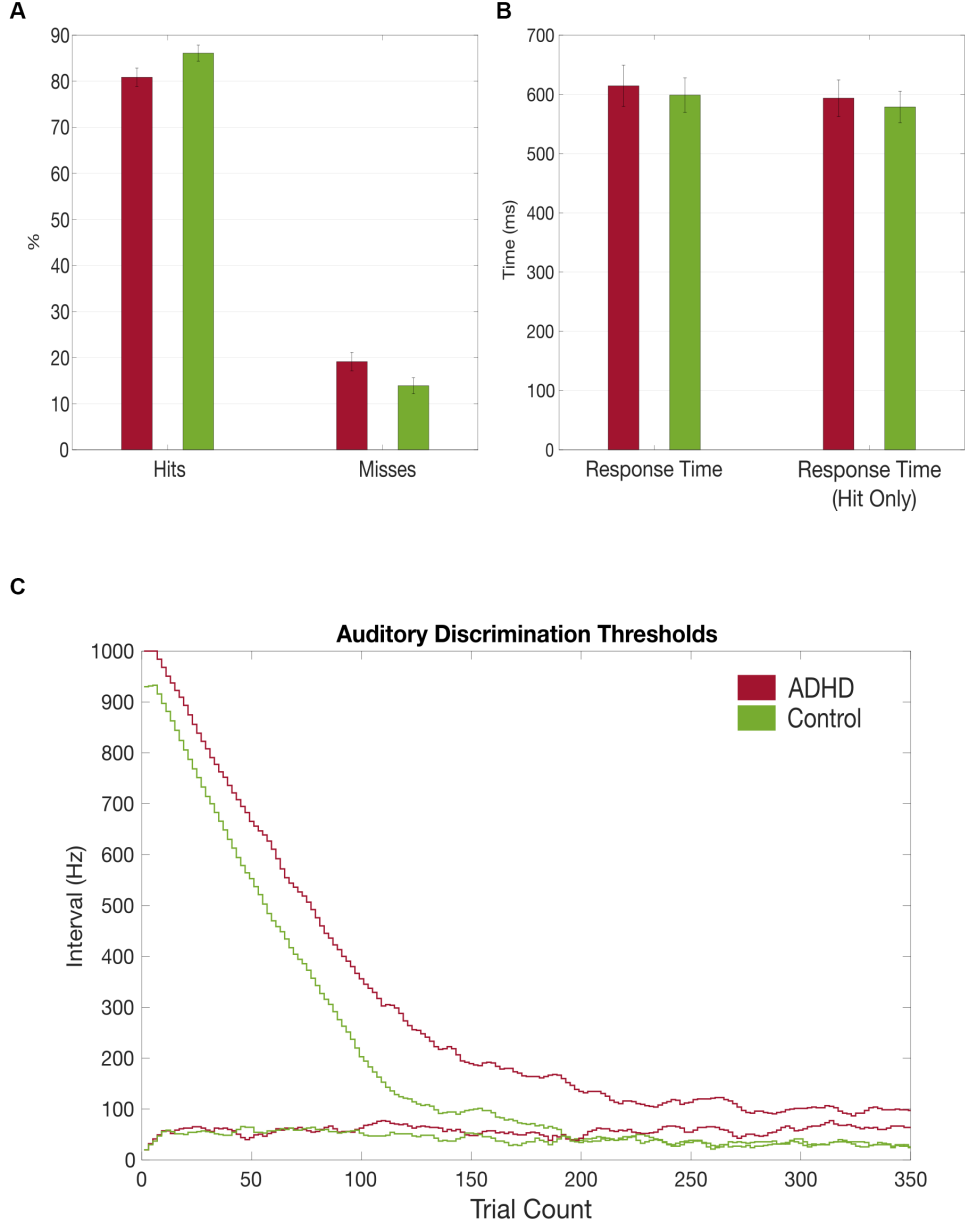
Grand average results of the auditory discrimination task. **(A)** Group comparison of correct (hits) vs. false (misses) answers in auditory discrimination task; **(B)** Group comparison of total auditory discrimination response times and response times (hit only) that included only correct answers; **(C)** Comparison of auditory discrimination thresholds between the patient (red) and control (green) group.

Although not reaching statistical significance, patients with ADHD discriminated the sounds’ frequency less often correctly [M_ADHD_ = *80.86% (SD = 7.70%)*, M_Controls_ = 86.01% (SD = 6.52%), *t* = −1.961, *p* = 0.06]. Miss rates were accordingly higher in patients with ADHD but not significant [M_ADHD_ = 19.1% *(SD = 7.68%)*, M_Controls_ = 13.9% (SD = 6.44%), *t* = 1.961, *p* = 0.06; Figure 3A]. Additionally, miss rates were compared between the first and the second half of the task and found to be significantly higher in the patient group in the first part [M_ADHD_ = 16.68% *(SD = 1.36%)*, M_Controls_ = 11.99% (SD = 1.50%), *t* = 2.11, *p* = 0.04], but not for the second half (*p* = 0.08). No within-group differences in misses were found between first half and second half if the trials. Patients with ADHD had overall higher auditory discrimination thresholds (38.67 vs. 13.57 Hz, *t* = 1.942, *p* = 0.06) and it took them more trials to reach their individual threshold (172.47 vs. 142.86, *t* = 1.425, *p* = 0.166; Figures 3A,B).

#### 3.2.2. Visual discrimination task

**Figure 4:**
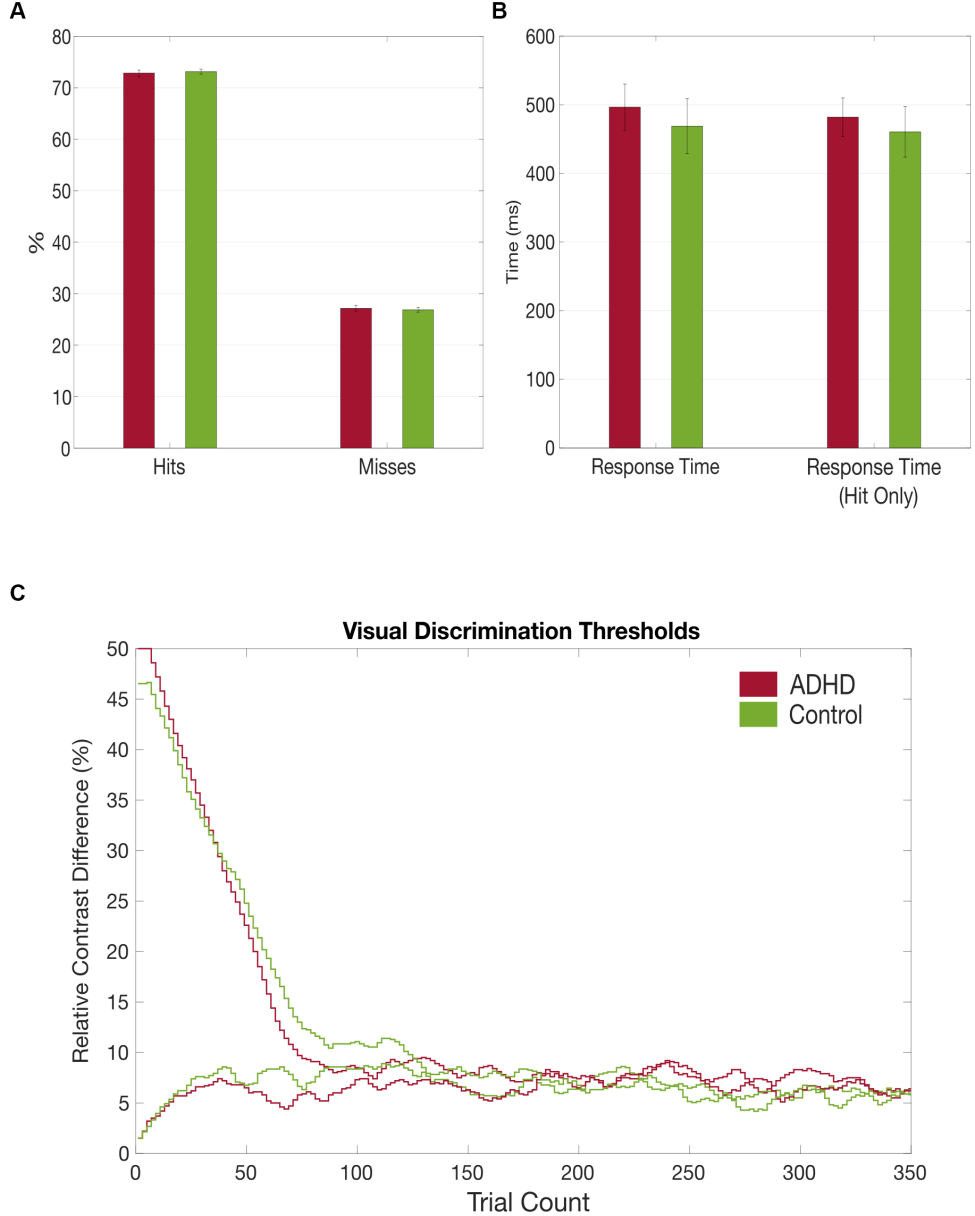
Grand average results of the visual discrimination task. **(A)** Group comparison of correct (hits) vs. false (misses) answers in visual discrimination task; **(B)** Group comparison of total visual discrimination response times and response times (hit only) that included only correct answers; **(C)** Comparison of visual discrimination thresholds between the patient (red) and control (green) group.

It took the patients longer to discriminate the gabor patches correctly compared to the healthy controls, despite also not reaching a statistical significance [M_ADHD_ = *496.52 ms (SD = 130.73 ms)*, M_Controls_ = 468.80 ms (SD = 149.99 ms), *t* = 0.466, *p* = 0.645]. Additionally, subjects with ADHD made more mistakes in discriminating the gabor patches’ contrast difference [M_ADHD_ = *27.16% (SD = 2.42%)*, M_Controls_ = 26.87% (SD = 1.86%), *t* = − 0.466, *p* = 0.645], although no statistical significance was reached too (see Table 5 and Figure 4). No group differences were found for misses between first and second half of the trials. However, both groups made significantly more mistakes in the second half of the trials than in the first [*t*_ADHD_ = −3.859 (*p* ≤ 0.01), *t*_Controls_ = −4.556 (*p* ≤ 0.01)].

### 3.3. EEG results

#### 3.3.1. Auditory discrimination task

##### 3.3.1.1. Intramodal ERPs

There were no significant group differences of P50, P100, N100, and P200 components at the channels Fz and Cz measured that represent intramodal auditory processing (Snyder et al., 2006). At the channel Fz, the N200 component amplitude showed to be significantly higher in the ADHD group than in the healthy controls [M_ADHD_ = *−1.08 (SD = 1.81)*, M_Controls_ = 0.76 (SD = 2.61), *t* = − 2.312, *p* = 0.04]. The details can be found in Table 2 and Figure 4.

##### 3.3.1.2. Crossmodal ERPs

**Figure 5:**
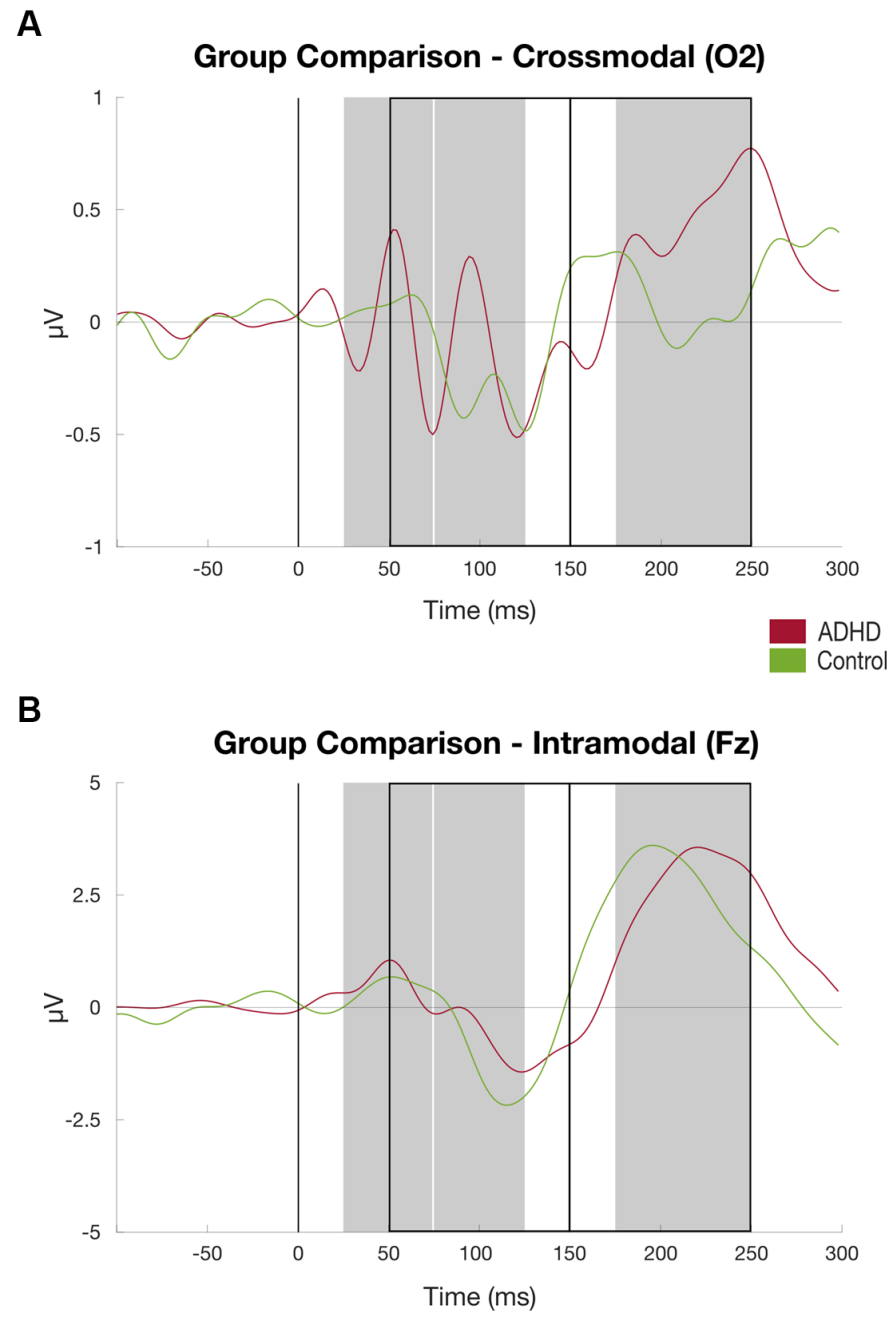
Results auditory discrimination task group comparison. **(A)** ERPs at channel O2 (crossmodal) averaged over subjects, patient group (red) vs. controls (green), time windows for subject-based peak detection as rectangles, and black line marks the stimulus onset (for details, see Figure 2); **(B)** Same as **(A)** at channel Fz.

The patient group elicited a significant higher mean amplitude found in the P200 component at the channel O2 compared to the healthy control group [*M_ADHD_ = 1.22 (SD = 0.73)*, M_Controls_ = 0.68 (SD = 0.48), *t* = 2.341, *p* < 0.05]. Additionally, the patient cohort showed higher activation, although not significant, at the channel O1 found in the P200 component compared to the control group [*M_ADHD_ = 1.00 (SD = 0.71),* M_Controls_ = 0.61 (SD = 0.64), *t* = 1.568, *p* = 0.128; see Table 2]. There were no further significant group differences of P50, P100, N100, and N200 components found at the channels O1 and O2 that represent crossmodal auditory processing (Figures 5).

**Figure 6:**
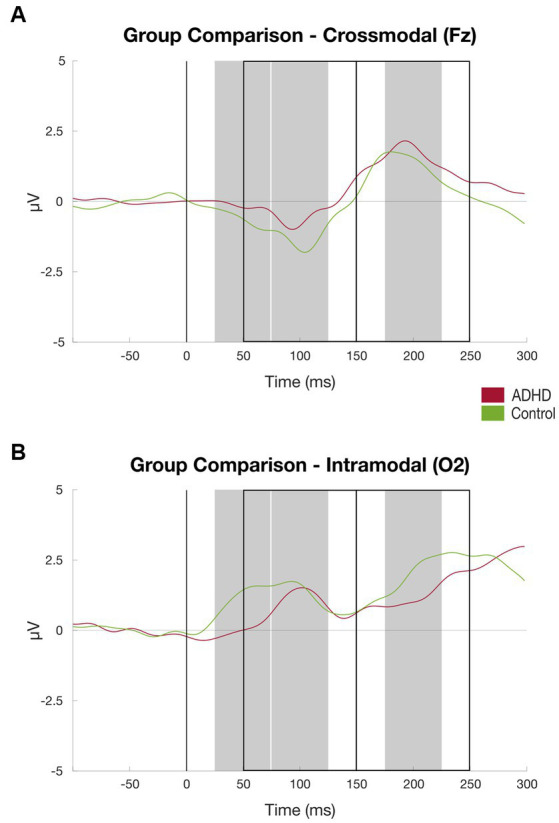
Results visual discrimination task group comparison. **(A)** ERPs at channel O2 (intramodal) averaged over subjects, patient group (red) vs. controls (green), time windows for subject-based peak detection as rectangles, and black line marks the stimulus onset (for details, see Figure 2); **(B)** Same as **(A)** at channel Fz (crossmodal).

#### 3.3.2. Visual discrimination task

##### 3.3.2.1. Intramodal ERPs

Compared to the healthy controls, the patient group elicited a significantly lower mean amplitude found in the P50 component at the channel O1 [*M_ADHD_ =* 0.56 (*SD =* 1.22), M_Controls_ = 2.30 (SD = 2.69), *t* = −2.247, *p* < 0.03] and channel O2 [*M_ADHD_ = 0.40 (SD = 1.10)*, M_Controls_ = 1.92 (SD = 2.44), *t* = −2.200, *p* < 0.03; see Table 3; Figure 6].

##### 3.3.2.2. Crossmodal ERPs

There were no significant group differences of P50, P100, P200, and N200 components at the channels Cz and Fz measured that represent crossmodal visual processing (Luck, 2014). At the channel Fz, the N100 component amplitude was significantly higher in the healthy controls than in the patient group [*M_ADHD_ =* − 1.59 (*SD =* 1.31), M_Controls_ = − 3.19 (SD = 1.64), *t* = 2.801, *p* < 0.01]. Details can be found in Table 3.

### 3.4. Association of sensory profiles/attention and auditory ERP components

In patients with ADHD sensation seeking behavior and the mean P200 amplitudes of the auditory discrimination task at the channel Fz was found to be significant negatively correlated, *r* = −0.517, *p* < 0.049. Further, the mean P100 amplitude and sensation seeking behavior were found to be positively correlated, *r* = 0.703, *p* < 0.05 in patients with ADHD. Visual crossmodal amplitude at the channel Fz was negatively correlated in the patients group (*r* = −59, *p* < 0.02). No significant association were found in the healthy control group.

## 4. Discussion

This is the first study addressing sensory crossmodal electrophysiological activity in adults with ADHD. In the auditory discrimination task, patients with ADHD showed a significantly enhanced crossmodal P200 amplitude and a significantly increased intramodal N200 amplitude compared to the healthy control group. No significant differences were found at earlier latencies. In the visual discrimination task, the patient group showed a significantly *lower* intramodal P50 at the channels O1 and O2 that represent intramodal sensory processing. At the channel Fz which represents crossmodal visual processing, the N100 component amplitude was significantly lower in the patient group compared to the healthy controls.

No other significant group differences of P50, P100, N100, P200, and N200 components were found. On the behavioral level, subjects with ADHD showed weaker auditory performances compared to the control group, although not reaching a statistical significance. In the visual discrimination task, the patient group also showed slightly but not significantly poorer performances.

Physiologically, the visual and auditory cortex are anatomically connected and influence each other already at early stages of sensory processing (Falchier et al., 2002; Molholm et al., 2002; Schroeder et al., 2003; Komura et al., 2005). The early crossmodal influence was found in the healthy control group at the crossmodal visual N100. This interaction is well measurable by a robust ERP component that is elicited in the visual cortex contralateral to a preceding laterally-presented sound after 250–400 ms. On the behavioral level, these spatially specific elicited sounds participate in visual perceptual alterations such as improved and faster discrimination, higher subjective intensity as well as the crossmodal reallocation of attentional resources to the visual domain (Retsa et al., 2020). In a study by Retsa et al., who investigated the conditions for the enhanced crossmodal visual activity to be measurable and concluded that it is dependent on the sounds’ spatial specificity. According to the authors, this dependency might indicate that early audiovisual crossmodal influences are only beneficial if the sound helps in selective visual attention processes, which are not required if the sounds are central and unspecific. Furthermore, it can be deduced that the suppression of crossmodal activations is equally important since they might lead to unnecessary spreading of attentional resources (Retsa et al., 2020).

In this study, we investigated auditory to visual crossmodal activations in ADHD by comparing the inter-group difference of the occipital P200 amplitude that was measured after the elicitation of a non-spatially specific sound. In general, the P200 component is associated with a number of early sensory processing steps including stimulus registration, discrimination, early attentive mechanisms as well as the inhibition of further processing of competing information (Näätänen, 1992; Korostenskaja et al., 2008; Barry et al., 2009; Lazzaro et al., 2009; Sur and Sinha, 2009; Stefanou et al., 2020). Until now, the P200 component has only been investigated at intramodal channels such as Fz and Cz in adults with ADHD (Micoulaud-Franchi et al., 2019), not at channels that can capture crossmodal activity. In the current study, the intramodal P50 and P200 component amplitudes between adults with ADHD and healthy controls did not differ significantly, which is in line with the results of Micoulaud-Franchi et al. (2019).

They were also able to show that the P50 component is related to later sensory processing, marked by a decreased P300 component that correlates with higher distractibility scores in the Sensory Gating Inventory. We found a higher intramodal auditory N200 and an enhanced crossmodal P200 component, indicating enhanced visual activity during the auditory discrimination task. This occipitally enhanced P200 component we measured in adults with ADHD can be related to the enhanced crossmodal visual activity in healthy populations that was mentioned before. One interpretation for the intergroup difference is that enhanced intra-and crossmodal auditory activity in ADHD reflects increased neuronal sensitivity in the processing of auditory stimuli and more sensory registration in the visual domain and thus more spreading of attentional resources, pointing toward deficient stimuli related inhibitory mechanisms in adults with ADHD. Our results are supported by the study of Salmi et al. (2018) who proposed that altered attentional processing in the auditory domain might result from enhanced visual activity.

The lack of significant ERP amplitude differences in the visual discrimination task indicates that early visual sensory processing of low saliency stimuli such as Gabor patches is not impaired in adults with ADHD. Still, there is evidence that visual processing does not function properly in patients with ADHD. While we investigated possible aberrancies in early stages of sensory processing, Cross-Villasana et al. (2015) measured delayed posterior contralateral negativity waves in adults with ADHD, indicating slower attentional selection in later stages of sensory processing. In addition to the impairment of unimodal visual processing in adults with ADHD, there is evidence that multisensory integration processes are aberrant as well, which were not investigated in this study (Schulze et al., 2020, 2021, 2022).

In the current study, subjects with ADHD had higher auditory discrimination thresholds, made more mistakes and it took them more trials to reach their minimal threshold compared to the healthy controls, despite not reaching a statistical significance (see Figures 3C,D). Fostick (2017) investigated auditory behavior in adults with ADHD by measuring their time order judgment (TOJ) thresholds and found that unmedicated adults with ADHD show significantly higher TOJ thresholds, whereas medicated patients had similar TOJ thresholds compared to the control group. This group difference of TOJ thresholds indicates (American Psychiatric Association, 2013) deficient auditory processing and (Sibley et al., 2017) that MPH might adjust the neuronal spreading to a similar degree as in healthy controls (Fostick, 2017). Additionally, Taitelbaum-Swead et al. (2019) compared auditory behavior between ADHD patients and healthy controls by measuring their listening effort in an audiovisual dual-task paradigm. They found that the ADHD group had lower accuracy in auditory attention when a visual distractor was present, which might be due to the deficient allocation of attentional resources to the auditory domain (Taitelbaum-Swead et al., 2019).

In the current study, adults with ADHD performed worse, although not significant, in the auditory discrimination task.

Besides the crossmodal spreading, one reason for the worse auditory performance might be impaired sustained attention capabilities in the patient group, as it is well reported as a common symptom in adult ADHD (Faraone et al., 2000; Marchetta et al., 2007). Subjects of the patient cohort scored significantly higher in the domain “hypersensitivity” compared to healthy controls, which is also in line with the literature (Faraone et al., 2000; Fostick, 2017; Panagiotidi et al., 2017). Although an auditory *hyper*sensitivity suggests better performances in auditory discrimination (Fostick, 2017), the underlying mechanisms that might explain the observed worse performance results are not yet disentangled. Possible Mechanisms for this observation are involuntary attentional shifts, crossmodal spreading of attentional resources as well as deficient inhibition mechanisms. As the results of other researchers show, early auditory processing deficits might indeed be a part of the pathophysiology of ADHD, although the performance differences we measured did not reach a statistical significance. It is therefore hard to make a definite deduction on the behavioral level and it is possible that there is no correlation between the significantly different EEG components and the task performances. Still, we observed that 10 of the 14 healthy subjects reached the smallest possible frequency interval between two tones after only 40% of all trials, which might be linked to a ceiling effect that results from the task design (see Table 5).

Further, higher sensory sensitivity goes along with less sensation seeking behavior, indicating lower sensory thresholds that lead to more auditory sensory registration. It is worth noting that the higher “low registration” scores (see Table 1) of the patient group do not contradict this enhanced auditory sensory registration, because the ASP results reflect sensory processing of all modalities, not just that of the auditory domain. This is in line with current studies that suggest a hypo- as well as hypersensitivity in different domains in people with ADHD (Kamath et al., 2020). Although our results do not show a significant group difference between the sensation seeking scores, we observed tendencies toward less sensation seeking behavior in subjects with ADHD (see Table 1).

The performance differences in the visual discrimination task between the groups are minor and therefore congruent with the lack of electrophysiological group differences. Both groups made more errors in the second half of the experiment, which can be interpreted considering increased difficultness for the contrast detection threshold. The lack of major effects strengthens the hypothesis that early visual sensory processing of low saliency stimuli like Gabor patches is not impaired in adults with ADHD. Still, there is evidence that aberrant visual processing leads to impaired visual performance in patients with ADHD, indicated by worse temporal allocation of visual attention (Hollingsworth et al., 2001), slower response times in a compound search task (Cross-Villasana et al., 2015), worse perception of depth, and higher distractibility (Panagiotidi et al., 2017). Though, these minor differences in visual processing, we found in our study may not result from early sensory processing, but from dysregulated top-down attentional processes. The performed tasks in our study need sustained attention, what is known to be regulated by dopaminergic and noradrenergic neuromodulation of the prefrontal cortex and impaired in adults with ADHD (Noudoost et al., 2010). Although rather speculative, future research is needed to disentangle the various influences of sensory processing aberrancies that lead to performance differences between people with ADHD and the neurotypical population.

### 4.1. Limitations

There are some limitations of this study worth noting. Although we recruited the patients by our outpatient department and the questionnaire results indeed suggest significant differences in ADHD traits and reflect characteristic sensory aberrancies that have shown to occur commonly in ADHD, it is possible that comorbidities may confound our findings. Further, we cannot rule out a possible medication influence despite having a wash-out period of at least 24 h. Additionally, the evaluation of the auditory task results might be limited through a ceiling effect, indicated by the fact that 10 of 14 healthy subjects reached the smallest possible frequency interval between two tones after only 40% of trials (see Table 5).

### 4.2. Conclusion

This is the first study to investigate early audiovisual crossmodal activations in adult ADHD using electrophysiology. The assessed difference in the enhanced auditory crossmodal P200 in subjects with ADHD can be interpreted as a marker for deficient inhibitory processes and more sensory registration, showing that there might be a distribution of attentional resources that participate in poorer unimodal auditory performances. One reason for those involuntary attentional shifts might be lower sensory thresholds that does not benefit subjects with ADHD, but leads to more impairment in their daily life through more inattention and higher distractibility. One potential next step that might help to understand the basic mechanism of impaired sensory processing in ADHD and their impact on symptomatology could be to study the interplay of crossmodal activation and attentional performance in ADHD.

## Data availability statement

The raw data supporting the conclusions of this article will be made available by the authors, without undue reservation.

## Ethics statement

The studies involving humans were approved by Kurt Racké, Ethical Committee Faculty of Medicine Bonn. The studies were conducted in accordance with the local legislation and institutional requirements. The participants provided their written informed consent to participate in this study.

## Author contributions

MiS: formal analysis, data curation, visualization, writing, and original draft preparation, MaS: conceptualization, formal analysis, project administration, and writing, review, and editing. TG: formal analysis and writing, review, and editing. BA: recruitment and writing, review, and editing. PJ: software and paradigm realization. SL and AP: supervision and writing, review, and editing. All authors contributed to the article and approved the submitted version.

## Funding

This work was supported by the Open Access Publication Fund of the University of Bonn.

## Conflict of interest

The authors declare that the research was conducted in the absence of any commercial or financial relationships that could be construed as a potential conflict of interest.

## Publisher’s note

All claims expressed in this article are solely those of the authors and do not necessarily represent those of their affiliated organizations, or those of the publisher, the editors and the reviewers. Any product that may be evaluated in this article, or claim that may be made by its manufacturer, is not guaranteed or endorsed by the publisher.
